# Assessing Vibrotactile Feedback Effects on Posture, Muscle Recruitment, and Cognitive Performance

**DOI:** 10.3390/s25082416

**Published:** 2025-04-11

**Authors:** Demir Tuken, Ian Silva, Rachel V. Vitali

**Affiliations:** 1 Department of Biomedical Engineering, University of Iowa, Iowa City, IA 52242, USA; demir-tuken@uiowa.edu; 2 Department of Mechanical and Electrical Engineering, University of Iowa, Iowa City, IA 52242, USA; ian-silva@uiowa.edu; 3 Department of Mechanical Engineering, University of Iowa, Iowa City, IA 52242, USA

**Keywords:** wearable sensors, inertial measurement unit, posture, spinal orientation, surface electromyography, muscle recruitment, haptic feedback, cognitive performance, ergonomics, musculoskeletal disorders

## Abstract

Musculoskeletal disorders are prevalent among medical professionals like dentists, who often maintain prolonged, ergonomically disadvantageous postures. This study aims to evaluate the feasibility and efficacy of a wearable sensor-based monitoring and feedback system designed to improve posture and evaluate muscle recruitment. Thirty-five healthy adults participated in a controlled experiment, performing a typing task under various postural conditions with and without haptic feedback. Surface electromyography sensors measured muscle activity in the upper trapezius and infraspinatus muscles, while inertial measurement units tracked spine orientation. The results indicated that haptic feedback significantly influenced muscle activity and posture. Feedback reduced deviations from the desired postures but increased muscle activity in certain conditions. Cognitive performance, measured by typing speed, decreased with feedback, suggesting a trade-off between maintaining posture and the performance of the task. These findings highlight the potential of haptic feedback in ergonomic interventions to mitigate MSDs. Future research should explore the long-term effects and optimize feedback mechanisms to balance posture correction and cognitive demands.

## 1. Introduction

Musculoskeletal disorders (MSDs) are non-acute conditions affecting muscles, connective tissue, joints, nerves, and blood vessels due to repetitive, prolonged mechanical loading [1,2]. They are the most common debilitating condition among working adults. Back pain from MSDs affects 300 million US workdays annually, equating to 8% of the workforce [2]. MSDs account for 30% of lost workdays, 50% of all days off work, and 33% of workers’ compensation costs in the US, resulting in USD 20 billion in direct costs and five times that in indirect costs [2]. Those with MSDs suffer from chronic pain and reduced quality of life, with medical professionals retiring 15 years early due to MSD injuries [3].

Healthcare workers, particularly those in surgical or dental fields, have a high risk of developing MSDs since many work tasks require them to maintain prolonged ergonomically disadvantageous or unsafe postures induced by environmental working conditions [4]. The associated chronic, debilitating pain significantly impacts their careers and lives, and it frequently leads to early retirement and, ergo, a critical loss of talent and productivity that required years of expensive training to obtain [5]. Even after retirement, these medical professionals often face prolonged recovery periods, continuing to deal with the lasting effects of MSDs for years. Previous work reported in the literature provides a wealth of subjective data, which seem to indicate a certain level of inaction given how few new worker safety regulations have been generated in recent decades in the face of the profuse prevalence and impact of these conditions.

The primary approach to assessing MSD prevalence among medical professionals is through surveys in which respondents self-report their physical condition. Social, psychological, and psycho-social questionnaires have also assessed perceived cognitive loads while working in pain [6,7]. One systematic review identified the neck, lower back, shoulder, and upper back to be the most reported areas of pain at 58%, 56%, 43%, and 41%, respectively, [8]. The amalgamated data suggest a strong relationship between working in the same position and discomfort while working. Another study identified the common causes for developing pain in the aforementioned areas, which include cervical flexion/rotation, elevated arms, and repetitive precise grips [9]. Other studies also note that static postures maintained for prolonged periods of time in concordance with bending, twisting, or repetitive motions can cause perceived MSD symptoms [10,11].

Although the problem is clear, an objective, quantifiable method to determine key factors or track improvements from interventions is missing [8]. This absence is evidenced by the limited number of quantitative studies reported in the literature focusing on the biomechanical behaviors exhibited by dentists. Finsen’s 1997 seminal field study examined the splenius and trapezius muscles of 12 practicing dentists, using surveys to link tasks with perceived pain [6]. Participants completed five dental tasks, but no significant differences in body segment positions or muscle activation were found. A 2016 study found significant differences in muscle activation between sitting and standing postures for splenius and sternocleidomastoideus muscles, but no change in asymmetries between postures was reported [12]. Another study assessed ergonomic dental supports, finding that magnification lenses, ergonomic stools, and their combination significantly reduced muscle activity in the upper trapezius, lateral deltoid, and anterior deltoid compared to having no support [13].

Feedback can promote ergonomic safety and can be delivered using different modalities. Visual feedback, such as a blinking light responding to muscle activation or body orientation, is effective for simple posture changes [14], but it may distract dentists during fine motor tasks. Audio feedback provides sound cues based on input parameters and helps retain muscle activity [15], but it may distract dentists who rely on auditory cues for patient comfort. In student nurses, auditory feedback achieved an approximate 7.5 degree reduction in trunk flexion [16]. Haptic feedback, using small (e.g., quarter-sized) vibrating motors, is widely used in healthcare ergonomic interventions due to its effectiveness without distraction [17]. It also promotes longer lasting behavioral changes compared to other feedback methods [18]. For example, the Simodont virtual reality dental training system uses concurrent haptic feedback to help students practice fine motor skills [17,19], which is effective for training students on how to complete the procedure but does not address ergonomic concerns. Only one study has investigated the effects of haptic feedback on the *biomechanical behavior* of practicing dentists. A 2015 study used a real-time feedback system with accelerometers and surface electromyography (sEMG) sensors to train dental students on posture [20]. The feedback reduced tilt angles and altered muscle activation, but the lack of normalized sEMG data limits the interpretation of these changes. Among pediatric otolaryngologists, vibrotactile biofeedback provided during surgery improved Rapid Upper Limb Assessment scores by 0.15 (95% CI: 0.05–0.25) [21].

The purpose of this study is to provide evidence for the feasibility and efficacy of a wearable sensor-based monitoring and feedback system for training the user to adopt specific postures. Here, participants engage in a simple typing task while maintaining different postures. Two approaches are utilized to ensure that participants maintain the desired posture as follows: (1) a physical guide that is removed prior to testing, and (2) a feedback system that provides haptic feedback. The data from the wearable sensors will be used to quantify differences in spine orientations and muscle activity. The specific hypotheses for this study include the following: (1) trials with an engaged posture have increased muscle activation than trials in a neutral posture, (2) deviations from a neutral sitting posture cause increased rates of muscle activation, (3) trials using feedback conform more to experimental testing configurations than trials without feedback, and (4) feedback decreases the user’s cognitive performance.

## 2. Materials and Methods

### 2.1. Participants

Thirty-five healthy able-bodied adults (height: 1.78 ± 0.13 m; weight: 78.7 ± 14.0 kg, 54.2 percent male) were recruited via mass email from the University of Iowa to participate in this study. Prior to testing, informed written consent was obtained from all participants involved in the study and approval was obtained from the University of Iowa Institutional Review Board. Participants who were sufficiently physically fit and in good health to maintain normal daily activity were enrolled to participate in the study. Prospective participants with any of the following conditions were excluded from the study: (1) muscle injuries that would limit physical activity, (2) recent fractures within the previous 6 months, (3) vestibular disorders that would affect balance, or (4) heart conditions that require recurring follow-up. Exclusion criteria were assessed through self-report via the recruitment email and reaffirmed verbally.

### 2.2. Experimental Procedure

**Figure 1:**
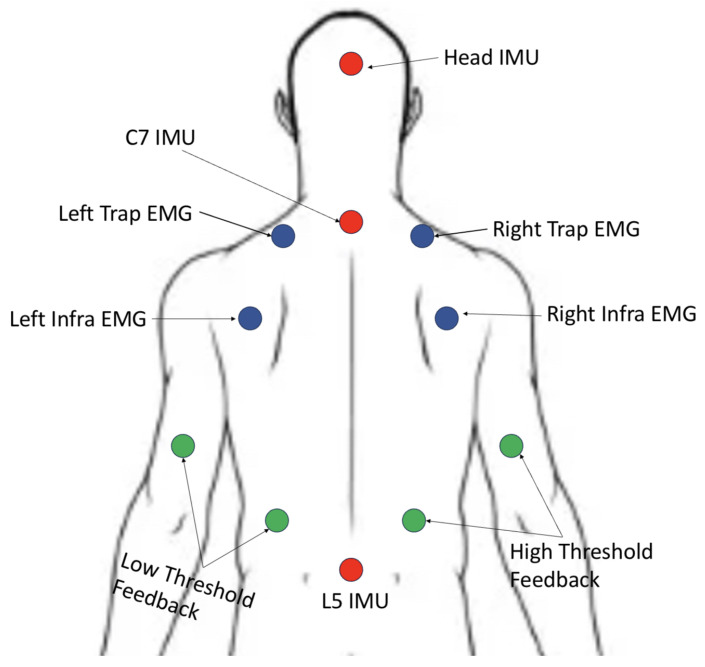
Placement of surface electromyography (sEMG) sensors (blue), inertial measurement units (IMUs; red), and haptic feedback devices (green).

The experimental protocol consisted of one 90-min, in-person visit conducted in a research laboratory. Participants reported their general demographics and information regarding their height, weight, and sex. Muscle activation was measured using sEMG Pico sensors (Cometa, Bareggio, Italy) [22]. The first target muscle is the infraspinatus, or one of the muscles located just below the shoulder blade that helps control the shoulder. The second target muscle is the upper trapezius, which spans much of the upper back just below the neck. Muscle activation was measured for both left and right muscles. Isometric maximum voluntary contractions (MVCs) were measured for each muscle following the surface EMG for a non-invasive assessment of muscles (SENIAM) protocol [23]. An additional 3 SageMotion inertial measurement units (IMUs) were taped on different parts of the participant’s spine to measure back and neck orientation (SageMotion, Kalispell, MT, USA) [24]. The IMUs were placed on the L5, C7, and posterior head positions and provide the means of estimating body segment orientation and, subsequently, the neck and back angles. Figure 1 illustrates the locations where the sensors were attached to each participant.

The design of the SageMotion IMUs also include vibrotactile motors that are capable of providing real-time haptic feedback (e.g., when the participant’s neck angle exceeds some threshold angular deviation). Four additional non-orientation sensing IMUs were placed on the participant that provide vibrotactile (haptic) feedback based on their posture, specifically the flexion and extension angles of the neck and back. The feedback sensor on the left (right) arm was used to indicate a low (high) neck angle. The sensor on the left (right) back was used to indicate a low (high) back angle. The feedback was delivered in this manner to be as descriptive as possible.

**Figure 2:**
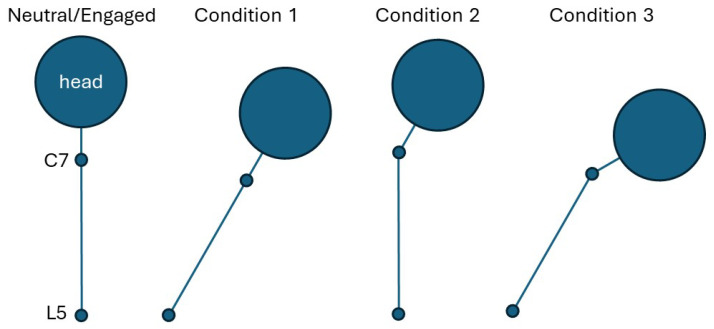
Illustrations of the conditions under which participants conducted the typing task.

To explore how feedback designed to alter posture affects muscle recruitment, each participant assumed the following pre-specified postures (see Figure 2) for an entire trial:

1. Neutral posture—the participant sits however is comfortable.
2. Engaged posture—the participant is instructed to sit upright without any back supports.
3. Condition 1: Back posture—back at 30° relative to vertical, head aligned with back.
4. Condition 2: Head posture—head at 30° relative to back aligned with vertical.
5. Condition 3: Combined back and head posture—head is at 30° relative to back and back at 30° relative to vertical.

The neutral and engaged postures were tested to determine an appropriate baseline with respect to muscle activation. Each condition had six trials, three with haptic feedback and three without feedback. Trials were conducted in a randomized order to prevent any learning effects with respect to incorporating the feedback to alter their spinal orientation.

During each trial, participants completed a words per minute (WPM) typing test [25] to capture cognitive performance. The WPM was recorded to collect quantitative data related to cognitive performance [26]. The WPM test was chosen to account for the inherent variability in typing skills among participants while establishing a standardized baseline to compare against for each participant to improve the interpretation of the results related to how haptic feedback influenced cognitive performance.

**Figure 3:**
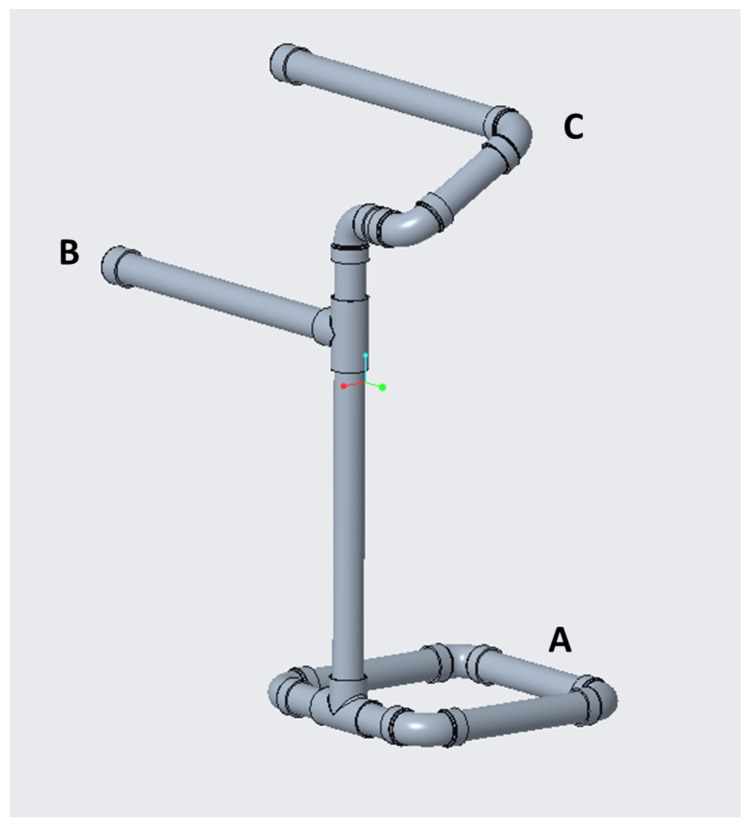
Experimental physical guide to assist in the initial posture setup. (A) The base which is kept in place with weights. (B) A hip guide that can be adjusted to the participant’s height. Participants are instructed to bend at the waist about the hip guide to assume the correct back angle. (C) The back and neck guide that is adjusted to help the participant assume the correct angle by touching their head to the guide. For Condition 3, the participant assumes the correct back angle and then neck angle, with a member of the research team adjusting the guide appropriately in between.

Prior to each trial in which the participant was instructed to assume Conditions 1, 2, and 3, an adjustable physical guide made of PVC piping (Figure 3) was used to help the participant understand how to orient their posture to adhere to each condition. The physical guide was configured to match the condition of the trial, and it was placed in front of the participant to help them conform to that condition. Once the participant was properly configured, it was removed before the trial began. Between each trial, participants rested for approximately 30–90 s while the adjustable physical guide was configured and put into place.

### 2.3. Surface Electromyography

The upper trapezius and infraspinatus muscles on the left and right side were located using the standard methods. Muscle testing sites were located by measurement and contractional palpation. The sEMG sensors were placed on the surface of each muscle. These sensors have a differential design and are 1 × 2.5 × 0.5 cm. To attach the sensors to the skin, Kendall 530 foam electrodes were used. Two electrodes were used per EMG sensor with each electrode measuring 1.5 inches in diameter. Prior to sensor placement, the skin was cleaned with isopropyl alcohol.

After electrode placement, participants warmed up the muscles of interest by contracting them three times at self-perceived levels of 20%, 50%, and 70%, relative to their maximal effort. It should be noted that participants were not provided with their muscle activation data during this process. For the infraspinatus muscles, the participant followed this warm-up guide leading up to 3 s with a 15 lb resistance band, with 1 min rest in between each exertion, repeated three times [27]. For the upper trapezius muscles, the participant followed this warm-up guide leading up to a 35 lb dumbbell shrug with both shoulders that was held for three seconds, with 1 min rest in between each exertion, repeated three times [28]. This protocol was used to establish the maximal voluntary contraction (MVC) for each muscle. For both MVC tests, the researcher team applied an opposite force to the participants motion. The weights were needed to assist in creating a maximum contraction. Reference contractions were also collected to compare against the maximum muscle contraction tests for validation. Verbal encouragement was also provided by the research team to help the participant reach their highest muscle activation in each trial.

The wireless Cometa sEMG sensors have a sampling frequency of 2000 Hz. Following a similar processing pipeline as [29], post-processing of the raw sEMG data included a bandpass filter, full wave rectification, and then a low pass filter. A fast Fourier transform (FFT) was utilized to inspect frequency content and was completed following the bandpass filtering. The cutoff frequencies for the bandpass filter were determined to be 25 Hz and 480 Hz. The cutoff frequency for the low pass filter was 20 Hz. Frequency cutoffs were also guided by previous studies focused on muscle activity data acquisition in workplace settings [20]. The sEMG data were then normalized to the highest post-processed reading in the MVC test, yielding the muscle activation percentages. To analyze the MVC-normalized sEMG data, the root mean square (RMS) was extracted to assess muscle activation. The 50-millisecond RMS segments captured a brief window of muscle activity to determine how the muscle activity changed over the duration of each trial.

### 2.4. Inertial Measurement Units

The L5 and C7 vertebrae were located by palpitation. IMUs were then taped on the L5, C7, and strapped to the posterior head position. The IMUs are equipped with built-in vibrotactile motors to produce vibratory feedback at 300 Hz. Four feedback sensors were used—two were placed on the lower back and two were placed on the arms. Each sensor was responsible for producing feedback for one condition as follows: low and high threshold for neck and back angles.

Each IMU includes a tri-axial accelerometer, angular rate gyroscope, and magnetometer that a proprietary Kalman filter uses to provide orientation estimates in quaternion form (see [30] for an example of a Kalman filter implementation). Once the sensor to world frame orientation was estimated, the sensor to body segment orientation needed to be determined. It was assumed the longitudinal axis of each body segment would be aligned with the direction of gravity during a known still period, which was calculated from the average accelerometer data that were then normalized to be a unit vector [31]. The sensor frame was then rotated such that one of the sensor frame axes was aligned with the normalized unit vector aligned with the longitudinal axis of the body segment. Then, the quaternions for two adjacent IMUs (head and C7 & C7 and L5) were multiplied to obtain the orientation of one body segment relative to the other. This relative orientation quaternion was then converted into an angle using the following equation to obtain the roll, as follows:

(1) $$\theta =arctan\left(t_{0},t_{1}\right)-\frac{\pi }{2}$$

where $t_{0}$ and $t_{1}$ are quaternion elements and subtracting the $90^{\circ }$ angle relates the sensor’s orientation to vertical [24]. The posture analysis was conducted in real time with Python 3.10.9, whereas the muscle activation analysis and all post-processing was conducted using MATLAB 2022a.

### 2.5. Statistical Analysis

To assess the significance of muscle activation in hypothesis one, a paired *t*-test was conducted to compare the average RMS of neutral postures to the average RMS of engaged postures. Cohen’s *d* was also calculated to evaluate the effect size associated with this comparison.

To assess the significance of muscle activation in hypothesis two, a linear mixed-effects regression (LME) model was used to compare the changes in muscle activity as a function of feedback and condition. Random effects of subjects were incorporated to account for individual differences. The average RMS of the neutral baseline was subtracted from the average RMS of each condition used in the LME model. The results from the paired *t*-test to assess hypothesis one supported using the neutral posture as the baseline when comparing each condition. Subtracting this baseline will give a better indication of the magnitude of changes in muscle activity induced by each postural condition. The dependent variable was the average RMS, $\bar{RMS}$, of each trial, and all tests were evaluated with a significance level of α=0.05. The LME model was as follows:

(2) $${\bar{RMS}}_{sEMG}∼1+Cond*Feed+\left(1|Subject\right)$$

The fixed effects include the intercept (‘1’), the trial condition for each typing task (*Cond*), and the feedback usage (*Feed*). The asterisk indicates the interaction between fixed effects is also included in the model. The random intercept is for participant ID (’1|*Subject*’). Each model was fitted for individual muscle groups.

To assess the significance of spine orientation in hypothesis three, the same LME model from Equation (2) was used to compare the changes in spine orientation as a function of condition and feedback with random participant effects included. For the posture analysis, the dependent variable was the average RMS difference, ${\bar{RMS}}_{diff}$, in the joint angle from the desired angle, and it was calculated for each trial. This model was evaluated for neck and back angles independently.

To assess the significant of cognitive performance in hypothesis four, changes were evaluated using the participants’ performance on the typing task. The difference in words per minute (WPM), $WPM_{diff}$, from the average words typed per minute in the neutral postures was calculated. This metric was then used as the dependent variable for the LME model described in Equation (2) to evaluate any changes in cognitive performance as a function of condition or feedback, while also allowing for individual differences through the random effect.

## 3. Results

**Figure 4:**
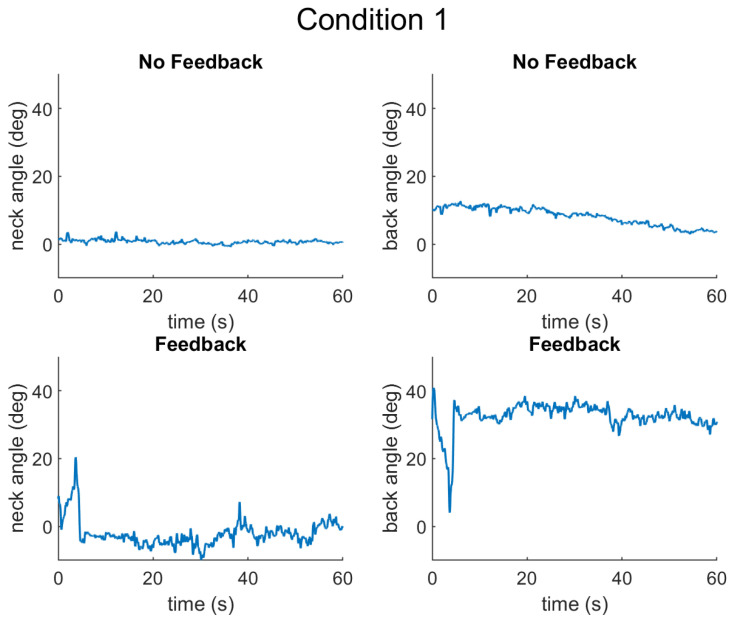
Neck and back angles for Condition 1, without and with feedback. In this condition, the back should be at a 30 degree angle, whereas the neck should be straight.

**Figure 5:**
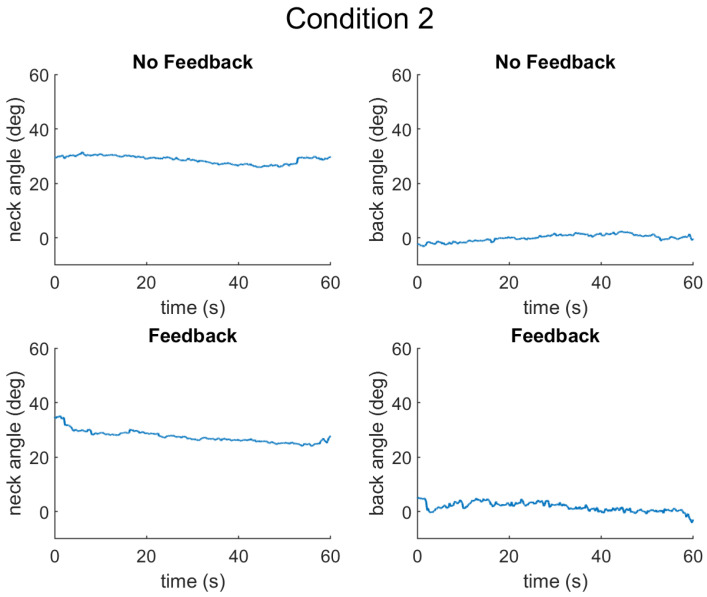
Neck and back angles for Condition 2, without and with feedback. In this condition, the neck should be at a 30 degree angle, whereas the back should be straight.

**Figure 6:**
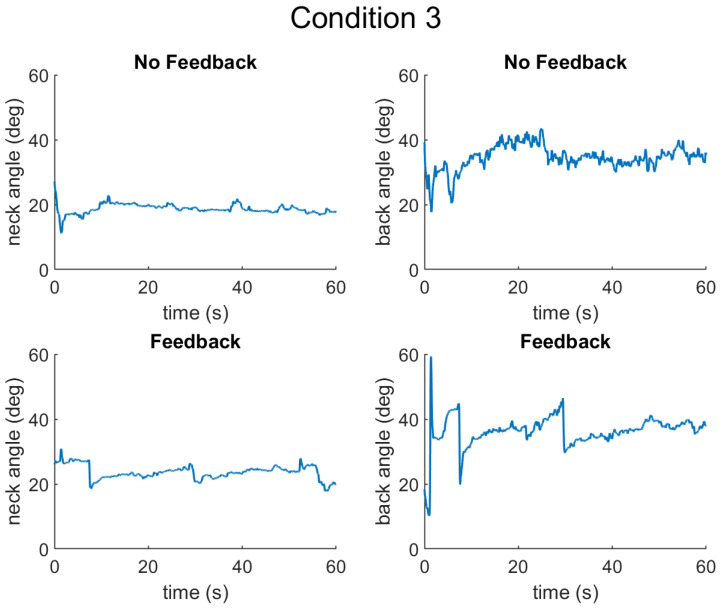
Neck and back angles for Condition 3, without and with feedback. In this condition, the neck and back should be at 30 degree angles.

The results are presented for each of the types of data collected as follows: (1) muscle activation measured by surface electromyography (sEMG) sensors, (2) posture measured by inertial measurement units (IMUs), and (3) cognitive performance measured by a typing task. First, the results from a representative participant are presented for Condition 1 (Figure 4), Condition 2 (Figure 5), and Condition 3 (Figure 6).

### 3.1. Muscle Activation Results

To assess the first hypothesis, a paired *t*-test evaluated the differences in muscle activity between neutral and engaged postures (Table 1). For reference, the thresholds for small, medium, and large effect sizes for Cohen’s *d* are 0.2, 0.5, and 0.8, respectively, [32]. The left trapezius showed a statistically significant decrease in muscle activity between neutral and engaged postures, though the effect size suggests this change is relatively small. On the other hand, the left infraspinatus does not have a statistically significant change, but it does exhibit a large effect size. This seemingly contrasting result indicates that participants responded differently, thereby creating large variation in the resulting muscle activity. Guided by these results, the neutral posture is used as the baseline for the subsequent muscle activity analyses.

**Figure 7:**
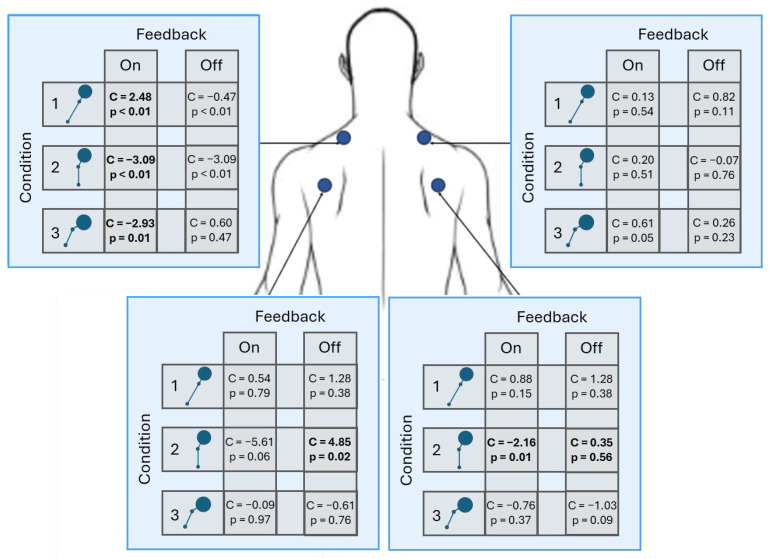
Visual summary of the statistical results reported in Table 2, Table 3, Table 4 and Table 5. The blue boxes in the upper left and right represent the LME coefficients for the left and right trapezius muscles. The blue boxes in the lower left and right represent the LME coefficients for the left and right infraspinatus muscles.

To assess hypothesis two, linear mixed effects (LME) models evaluated each muscle group compared to a neutral posture baseline, a summary of which is illustrated in Figure 7.

Table 2 and Table 3 report the LME results for the right and left trapezius muscles, respectively. The results indicate that feedback and condition type can induce changes in muscle activity. To aid in interpretation, Table A1 and Table A2 report the predicted values for each combination of the fixed main and interaction effects for muscle activation for these two types of muscles. In Condition 1, the presence of feedback coincided with an increase in muscle activity for both. For the other two conditions, feedback usage increased muscle activity in the right trapezius and decreased muscle activity in the left trapezius.

The LME regression results for the infraspinatus are reported in Table 4 and Table 5. The results continue to show feedback and condition type can induce changes in muscle activity.

To aid in interpretation, Table A3 and Table A4 report the predicted values for each combination of the fixed main and interaction effects for muscle activation for these two muscles. Similar to the trapezius muscles, the presence of feedback coincided with an increase in muscle activity for both infraspinatus muscles in Condition 1. In both infraspinatus muscles, feedback usage induced decreases in muscle activity in Condition 2 and increases in muscle activity in Condition 3. Interestingly, the left infraspinatus is the only muscle with a statistically significant different level of muscle activity when feedback is not present (i.e., Condition 2).

### 3.2. Posture Results

**Figure 8:**
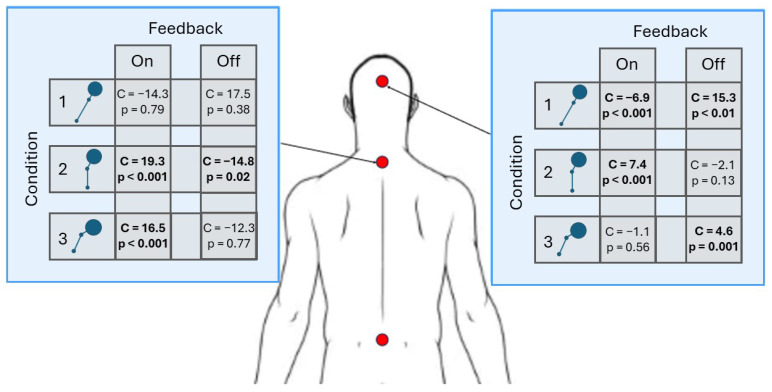
Visual summary of the statistical results reported in Table 6 and Table 7. The blue box on the left contains the LME results for the back angles. The blue box on the right contains the LME results for the neck angles.

Next, the posture data were analyzed using an LME model to assess hypothesis three, as summary of which is illustrated in Figure 8 below. Table 6 and Table 7 report the full statistical results for the neck and back angles, respectively. As a reminder, the joint angles are compared to the desired joint angle for the specific condition (i.e., either $0^{\circ }$ or $30^{\circ }$).

To aid in interpretation, Table A5 and Table A6 report the predicted values for each combination of the fixed main and interaction effects for changes in neck and back angles, respectively. For Condition 1, the deviations in both the neck and back angle decreased in deviation from the desired posture in the presence of feedback. Conversely, the deviations in both angles increased when feedback was introduced in Condition 2. While deviations decreased for neck angles with feedback for Condition 3, the deviations increased for back angles.

### 3.3. Cognitive Performance Results

For the cognitive performance results, the random effect due to subject was determined to be statistically insignificant. Thus, the analysis was conducted again with the random effect removed, the results of which are reported in Table 8. To assess hypothesis four, there was a decrease in average words per minute compared to the neutral baseline when feedback was present, which was more prevalent in Conditions 2 and 3 than in Condition 1.

## 4. Discussion

Overall, the findings suggest that inducing a change in posture to complete a task is possible with haptic feedback, but this will also affect the user’s physiological response to said change. It is also important to consider how the participants are instructed, especially when establishing a baseline. Considering hypothesis one, the left trapezius produced a significant change in muscle activity for engaged postures when compared to neutral postures. As the Cohen’s *d* was negative for the left trapezius, the engaged posture produced lower muscle activity than the neutral posture. However, the large Cohen’s *d* for the left infraspinatus indicates the engaged posture induced a large (positive) change in muscle activity, even though the *p*-value was insignificant. This result indicates that there was no consistent change in muscle activity when comparing an engaged posture to neutral postures for a simple typing task. These findings are important in creating a basis for understanding the different changes in muscle activity for these two postures. Both produce different biomechanical loading patterns on the muscles, which results in varying muscle activity, as is evident from the results.

With respect to hypothesis two, there were some slight increases in muscle activity in the absence of feedback, with a few exceptions. For instance, the left trapezius and right infraspinatus exhibited a decrease in muscle activity in Conditions 1 and 3, respectively. However, both decreases failed to reach statistical significance. On the other hand, the left infraspinatus exhibited a statistically significant increase, but only for Condition 2. Thus, there is some slight evidence supporting the second hypothesis. The decrease in muscle activity was unexpected. The literature that examined muscle activities for dental working tasks saw nonsignificant increases in muscle activity compared to normal working procedures [12]. The majority of changes in muscle activity occurred when feedback was present, largely for the left trapezius. However, the changes were not consistent, which makes it difficult to ascertain generalizations about how the feedback induced changes based on the results of this study. For Condition 1, feedback increased muscle activity for all four muscle groups. For the other two conditions, the muscle activity increased and decreased for different muscle groups. While most of the results presented here agree with the previous literature, the significant decreases in muscle activity provide a new avenue for evaluating muscle loading patterns during task completion.

With respect to hypothesis three, there were significant changes in both neck and back angles with and without feedback. On average, the neck angles would deviate 17.6° from the desired angle without feedback. When feedback was introduced, those deviations reduced to 12.8°, on average, or a 27% improvement was demonstrated. For the back angles, the average deviations without feedback were 8.5°, which decreased with feedback to 6.1° or showed a 27% improvement. When considering the averages with and without feedback (and the significant main effect of feedback), there is support for this hypothesis. However, these averages ignore the dependence on the condition. For example, in Condition 1, both angle deviations decreased with feedback. However, for Condition 2, both angle deviations slightly increased with feedback. Furthermore, for Condition 3, the neck angle deviations decreased while the back angle deviations slightly increased.

To help explain these results, let us now consider the cognitive performance results and hypothesis four. All conditions with feedback saw a significant decrease in average words per minute typed, while none of the conditions without feedback were significantly different from the baseline. These results were somewhat expected as a few participants anecdotally shared that interpreting the four different haptic feedback devices was occasionally challenging. The feedback, in its present form, essentially transformed the simple typing task into a complex one, during which the participants were forced to focus more on how to alter the orientation of their spine to adhere to the feedback. This complexity may also contribute to participants being able to conform to one of the anatomical angles more easily than the other. For example, Condition 1 required the participants to maintain a 30° neck angle and an upright back angle. The feedback helped participants reduce their deviations, but this was at the cost of increasing muscle activity. This behavior may be explained by the participants tensing their muscles to maintain a more rigid posture than they could readily maintain. In Condition 2, the participants maintained a 30° back angle and kept their head aligned with their back. Here, the feedback slightly increased their deviations, while muscle activity in three of the four muscles decreased. This behavior may be explained by the participants relaxing their muscles to maintain a less rigid posture than they were seeking to assume. Finally, in Condition 3, the participants maintained a 30° neck and back angle. Feedback helped participants reduce their neck angle deviation but hindered their ability to reduce their back angle deviation. This disparity is highlighted further by the fact that participants increased their muscle activity in three of the four muscles. Given the challenge of interpreting the feedback, participants may have focused on maintaining one of the spinal orientations (i.e., the neck) in which they conformed more to their behavior in Condition 1. Future work should carefully consider the complexity of the feedback being delivered to the user.

The introduction focused on the prevalence of MSDs among healthcare workers due to prolonged, ergonomically disadvantageous postures. Previous studies have shown that feedback, whether visual, auditory, or haptic, can promote ergonomic safety. Haptic feedback, in particular, has been shown to be effective, without causing too much distraction, and this study’s results are consistent with these characteristics. The haptic feedback used in this study significantly influenced muscle activity and posture, reducing deviations from desired postures but increasing muscle activity in certain conditions. These findings are in line with the study by Finsen (1997) [6], which found no significant differences in muscle activation between different dental tasks, and the study by Al-Qaisi and Aghazadeh (2015), which highlighted the importance of feedback in maintaining ergonomic postures [28].

One of the key gaps identified in the literature is the lack of information on how haptic feedback designed to alter posture affects muscle activation. This study contributes to filling this gap by providing empirical evidence on the effects of haptic feedback on muscle activity and posture. The results show that while haptic feedback can help maintain the desired postures, it can also lead to increased muscle activity. This finding is crucial, as it highlights the potential trade-offs between posture correction and muscle activation, which have not been extensively explored in previous studies.

### Current Limitations

This study examined a total of 24 testing minutes for each participant, with rest periods between each 1-min trial. Using the load-tolerance concept as guidance [11], tolerance decreases as time progresses when a muscle group undergoes cyclical loading. Without studying long term feedback usage, these results cannot provide evidence of whether feedback changes the tolerance over time for participants. It should be noted that isolating the infraspinatus muscle can be difficult. Despite efforts to ensure that this relatively deep muscle was isolated, it is possible that the infraspinatus sEMG data could also contain crosstalk from the surrounding muscle groups. Future work should consider this difficulty prior to collecting muscle activation data from this muscle. Furthermore, the MVC protocols here instructed participants to maintain their maximum effort for 3 s, but the recent literature recommends having participants maintain the poses for 5 s or more (see, for example, [33]).

Future work is recommended to consider the intra- and inter-subject variability in defining the anatomical frames using inertial measurement units. Furthermore, it is possible that gender differences and/or anthropometric differences may affect how participants respond to haptic feedback to correct posture. The handedness was also collected as demographic information, which might aid in explaining the asymmetrical differences in muscle activity. While not considered here, future work should also consider the effects of coactivation between agonist–antagonist muscles, which could potentially shed some light on how participants are altering their muscle recruitment behavior in response to the haptic feedback input.

## 5. Conclusions

The purpose of this study was to evaluate the feasibility and efficacy of the wearable sensor-based monitoring and feedback system by quantifying differences in spine orientations and muscle activity. Participants completed a simple typing task in three different postures, with and without feedback present, to guide their body segment orientations. Only one muscle, left trapezius, displayed a significant negative change in muscle activity between neutral and engaged postures, which was unexpected. The results show how haptic feedback designed to regulate posture can affect muscle activity, sometimes advantageously, though sometimes detrimentally. For the main effect of the condition, only one muscle, left infraspinatus, significantly increased its activation. When feedback was introduced, muscle activation increased and decreased, usually depending on the posture being assumed. The decreases in muscle activity provide a new perspective on evaluating muscle loading patterns, which could lead to novel approaches in ergonomics and occupational health to reduce the risk of work-related MSDs. On average, the feedback was able to reduce joint angle deviations, though in one condition it slightly increased the deviations. These somewhat conflicting results can be partially explained by the decrease in cognitive performance in the presence of feedback, indicating the participants were distracted by interpreting and implementing the feedback. This finding could have implications for the design and effectiveness of concurrent feedback-based interventions.

## Figures and Tables

**Table 1:** Summary of paired *t*-test to compare the average EMG RMS for neutral and engaged postures. Cohen’s *d* test was completed for post hoc analysis.

Muscle Group	*p*-Value	Cohen’s *d*
**Right Trapezius**	0.35	−0.21
**Right Infraspinatus**	0.32	−0.16
**Left Trapezius**	0.048	−0.35
**Left Infraspinatus**	0.22	0.79

**Table 2:** LME regression results for the average RMS of the right trapezius. CI:LB and CI:UB denote the lower and upper bounds of the 95% confidence intervals.

	Coefficient	*p*-Value	CL:LB	CL:UB
**Main Fixed Effects**				
**(Intercept)**	0.82	0.11	−0.21	1.86
**Condition 2**	−0.07	0.76	−0.51	0.37
**Condition 3**	0.26	0.23	−0.17	0.70
**Feedback**	0.13	0.54	−0.30	0.57
**Fixed Interaction Effects**				
**Condition 2: Feedback On**	0.20	0.51	−0.41	0.82
**Condition 3: Feedback On**	0.61	0.05	−0.004	1.24
**Random Effects**				
**Subject**	2.67	-	2.04	3.48

**Table 3:** LME regression results for the average RMS of the left trapezius. CI:LB and CI:UB denote the lower and upper bounds of the 95% confidence intervals.

	Coefficient	*p*-Value	CL:LB	CL:UB
**Main Fixed Effects**				
**(Intercept)**	−0.47	0.61	−2.34	1.39
**Condition 2**	0.78	0.35	−0.86	2.42
**Condition 3**	0.60	0.47	−1.03	2.24
**Feedback**	2.48	**<0.01**	0.84	4.12
**Fixed Interaction Effects**				
**Condition 2: Feedback On**	−3.09	**<0.01**	−5.42	−0.77
**Condition 3: Feedback On**	−2.93	**0.01**	−5.25	−0.61
**Random Effects**				
**Subject**	3.94	-	2.95	5.26

Bolded entries indicate significance at α = 0.05.

**Table 4:** LME regression results for the average RMS of the right infraspinatus. CI:LB and CI:UB denote the lower and upper bounds of the 95% confidence intervals.

	Coefficient	*p*-Value	CL:LB	CL:UB
**Main Fixed Effects**				
**(Intercept)**	0.41	0.33	−0.43	1.26
**Condition 2**	0.35	0.56	−0.84	1.55
**Condition 3**	−1.03	0.09	−2.23	0.16
**Feedback**	0.88	0.15	−0.32	2.08
**Fixed Interaction Effects**				
**Condition 2: Feedback On**	−2.16	**0.01**	−3.86	−0.46
**Condition 3: Feedback On**	−0.76	0.37	−2.46	0.93
**Random Effects**				
**Subject**	3.96	-	3.72	4.21

Bolded entries indicate significance at α = 0.05.

**Table 5:** LME regression results for the average RMS of the left infraspinatus. CI:LB and CI:UB denote the lower and upper bounds of the 95% confidence intervals.

	Coefficient	*p*-Value	CL:LB	CL:UB
**Main Fixed Effects**				
**(Intercept)**	1.28	0.38	−1.60	4.17
**Condition 2**	4.85	**0.02**	0.77	8.93
**Condition 3**	−0.61	0.76	−4.69	3.47
**Feedback**	0.54	0.79	−3.54	4.62
**Fixed Interaction Effects**				
**Condition 2:Feedback On**	−5.61	0.06	−11.38	0.16
**Condition 3:Feedback On**	−0.09	0.97	−5.86	5.68
**Random Effects**				
**Subject**	13.46	-	12.66	14.32

Bolded entries indicate significance at α = 0.05.

**Table 6:** LME regression results for average neck angle deviation. CI:LB and CI:UB denote the lower and upper bounds of the 95% confidence intervals.

	Coefficient	*p*-Value	CL:LB	CL:UB
**Main Fixed Effects**				
**(Intercept)**	15.3	**<0.01**	4.9	22.9
**Condition 2**	2.1	0.13	−0.8	3.7
**Condition 3**	4.6	**0.001**	3.2	5.1
**Feedback**	−6.9	**<0.001**	−9.2	−2.3
**Fixed Interaction Effects**				
**Condition 2:Feedback On**	7.4	**<0.001**	3.1	8.1
**Condition 3:Feedback On**	−1.1	0.56	−3.1	1.3
**Random Effects**				
**Subject**	9.7	-	6.8	10.3

Bolded entries indicate significance at α = 0.05.

**Table 7:** LME regression results for average back angle deviation. CI:LB and CI:UB denote the lower and upper bounds of the 95% confidence intervals.

	Coefficient	*p*-Value	CL:LB	CL:UB
**Main Fixed Effects**				
**(Intercept)**	17.5	0.38	−0.3	19.9
**Condition 2**	−14.8	**0.02**	−16.2	−1.2
**Condition 3**	−12.3	0.77	−18.2	2.6
**Feedback**	−14.3	0.79	−15.3	12.5
**Fixed Interaction Effects**				
**Condition 2:Feedback On**	19.3	**<0.001**	17.2	21.5
**Condition 3:Feedback On**	16.5	**<0.001**	11.3	17.4
**Random Effects**				
**Subject**	10.5	-	9.9	11.9

Bolded entries indicate significance at α = 0.05.

**Table 8:** Linear regression results for the average words per minute. CI:LB and CI:UB denote the lower and upper bounds of the 95% confidence intervals.

	Coefficient	*p*-Value	CL:LB	CL:UB
**Main Fixed Effects**				
**(Intercept)**	3.2	0.79	−1.4	4.8
**Condition 2**	0.7	0.08	−0.3	2.2
**Condition 3**	0.9	0.44	−6.2	9.0
**Feedback**	−3.3	**<0.01**	−5.9	−2.1
**Fixed Interaction Effects**				
**Condition 2: Feedback On**	−2.0	**0.04**	−4.1	−1.3
**Condition 3: Feedback On**	−2.1	**<0.01**	−3.2	−2.0

Bolded entries indicate significance at α = 0.05.

## Data Availability

The de-identified datasets may be available upon reasonable request.

## Appendix A

### Appendix A.1. Predicted Muscle Activation Values from Linear Mixed Effects Analyses

These tables document the predicted values for each combination of the fixed main and interaction effects for muscle activation results based on the linear mixed-effects analysis.

**Table A1:** Predicted differences in right trapezius muscle activation relative to the baseline, using the results from the linear mixed-effects regression model described in Table 2.

Feedback	Condition 1	Condition 2	Condition 3
OFF	0.82	0.75	1.08
ON	0.95	1.08	1.82

**Table A2:** Predicted differences in left trapezius muscle activation relative to the baseline, using the results from the linear mixed-effects regression model described in Table 3.

Feedback	Condition 1	Condition 2	Condition 3
OFF	−0.47	0.31	0.13
ON	2.01	−0.30	−0.32

**Table A3:** Predicted differences in right infraspinatus muscle activation relative to the baseline, using the results from the linear mixed-effects regression model described in Table 4.

Feedback	Condition 1	Condition 2	Condition 3
OFF	0.41	0.76	−0.62
ON	1.29	−0.52	−0.50

**Table A4:** Predicted differences in left infraspinatus muscle activation relative to baseline, using the results from the linear mixed-effects regression model described in Table 5.

Feedback	Condition 1	Condition 2	Condition 3
OFF	1.28	6.13	0.67
ON	1.82	1.06	1.12

### Appendix A.2. Predicted Joint Angle Values from Linear Mixed Effects Analyses

These tables document the predicted values for each combination of the fixed main and interaction effects for the joint angle results based on the linear mixed-effects analysis.

**Table A5:** Predicted differences in neck angle relative to the baseline, using the results from the linear mixed-effects regression model described in Table 6.

Feedback	Condition 1	Condition 2	Condition 3
OFF	15.3	17.4	19.9
ON	8.4	17.9	11.9

**Table A6:** Predicted differences in back angle relative to the baseline, using the results from the linear mixed-effects regression model described in Table 7.

Feedback	Condition 1	Condition 2	Condition 3
OFF	17.5	2.7	5.2
ON	3.2	7.7	7.4

### Appendix A.3. Predicted Cognitive Performance Values from Linear Regression Analysis

This table documents the predicted values for each combination of the fixed main and interaction effects for the words per minute typed by the participant based on the linear regression analysis.

**Table A7:** Predicted differences in words per minute typed to the baseline, using the results from the linear regression model described in Table 8.

Feedback	Condition 1	Condition 2	Condition 3
OFF	3.2	3.9	4.1
ON	−0.1	−1.4	−1.3
